# Rapid Estimation of Potato Quality Parameters by a Portable Near-Infrared Spectroscopy Device

**DOI:** 10.3390/s21248222

**Published:** 2021-12-09

**Authors:** Olga Escuredo, Laura Meno, María Shantal Rodríguez-Flores, Maria Carmen Seijo

**Affiliations:** Department of Vegetal Biology and Soil Sciences, Faculty of Sciences, University of Vigo, 32004 Ourense, Spain; laura.meno@uvigo.es (L.M.); mariasharodriguez@uvigo.es (M.S.R.-F.); mcoello@uvigo.es (M.C.S.)

**Keywords:** NIR spectrometer, intact potato, dry matter, reducing sugars, chemometrics, MPLS

## Abstract

The aim of the present work was to determine the main quality parameters on tuber potato using a portable near-infrared spectroscopy device (MicroNIR). Potato tubers protected by the Protected Geographical Indication (PGI “Patata de Galicia”, Spain) were analyzed both using chemical methods of reference and also using the NIR methodology for the determination of important parameters for tuber commercialization, such as dry matter and reducing sugars. MicroNIR technology allows for the attainment/estimation of dry matter and reducing sugars in the warehouses by directly measuring the tubers without a chemical treatment and destruction of samples. The principal component analysis and modified partial least squares regression method were used to develop the NIR calibration model. The best determination coefficients obtained for dry matter and reducing sugars were of 0.72 and 0.55, respectively, and with acceptable standard errors of cross-validation. Near-infrared spectroscopy was established as an effective tool to obtain prediction equations of these potato quality parameters. At the same time, the efficiency of portable devices for taking instantaneous measurements of crucial quality parameters is useful for potato processors.

## 1. Introduction

The potato is a traditional crop and the base of human diet in many world regions. In the past two decades, potato production has experienced a greater growth compared with other tubers, due to its high yield and human nutritional value [1,2]. This increase is favored by the need to meet the increased demand for food due to the world population growth [2]. Undoubtedly, the potatoes have played an important role in food availability, and are currently still holding this role.

The potatoes are characterized as a good source of starch with unique characteristics compared with the basic starches of cereals. In addition to fresh consumption, tubers can be destined for the processing industry (such as chips, flakes, dry and frozen potatoes), for additional food ingredients, such as tuber seed for field cultivation, animal feed, and in the chemical industry as a source of starch and ethanol [2,3]. The potato in Spain is mainly intended for fresh consumption, industrial processing, and animal feed. The highest production of this tuber is found in the Northwest of Spain. Specifically, in Galicia, this traditional agricultural activity is an important source of economic income for families [4], in order for the production to be covered by the Protected Geographical Indication (PGI) Patata de Galicia [5]. Kennebec, Agria, and Fina de Carballo are the potato varieties, which are protected by the designation of origin comprising the largest extensions of this crop in Galicia.

The potato tubers are stored for several months after their harvest, in order to meet the market demands throughout the year. This increases their marketability and generates an additional benefit for growers, processors, and consumers [6,7]. The composition of the potato varies with the storage time and cultivar type [7,8,9]. Generally, the respiration and the evaporation rate increase during maturity and tuber storage. This produces an increase in weight loss and peroxidase enzymatic activity together with a decrease in starch and ascorbic acid content. As a consequence, reducing sugars are synthesized [8,10,11]. Therefore, the nutritional quality degradation of potato during their storage is induced, with changes in starch content, dry matter, and reducing sugars [7,8,12]. Reducing sugar is an important indicator for evaluating the quality of raw material in the processing of potato industry [13]. Excess levels of reducing sugars cause an unacceptable non-enzymatic brown color for fried products, due to the reaction between the reducing sugars and the α-amino groups of amino acids [6,13,14]. Therefore, high reducing sugars in tubers are not suitable for processing.

The industry has innovated and invested in improved post-harvest storage, preserving potato quality for the seed, fresh, and processing sectors [3,15]. The acceptance of the tuber in fresh markets depends mainly on its external appearance [9], but with dependence on the internal composition, which is determined by destructive analytical procedures. The amount of dry matter and reducing sugars are the main physicochemical parameters that determine the industrial yield, quality, and flavor of potato tubers. The most used conventional analytical techniques are colorimetric and titration measurement methods [13]. In addition, the operators themselves based on their professional experience select the potato tubers by hand on the processing belt. However, this operation is not enough to guarantee optimal quality and compliance with quality standards. Analytical methods commonly employed to determine the main compounds of potatoes do not seem to be suitable for in-line applications in the food industry since they require a large amount of time and are destructive. Therefore, ensuring the minimum level of quality of basic foods that is accepted by the consumer requires assessing its quality by swift and non-destructive techniques [6,16,17]. As a result, the importance of quantifying the dry matter and the sugars in-line during potato processing ensures optimal quality and discards unsuitable tubers for marketing.

In the last years, visible- and near-infrared (VIS-NIR) spectroscopy has contributed to providing non-destructive methods for the evaluation of the internal quality of fresh fruits and vegetables or cereals [7,17,18,19,20]. The advantages of NIRS are time saving, offering the ability to record many quality characteristics or ingredients with a single measurement. Some studies have been conducted to test the near-infrared spectroscopy measuring quality parameters of potatoes, such as sugars or dry matter content in laboratory [6,12,14,17,21,22,23,24,25]. According to some researchers, the results of these studies are difficult to compare since some are focused on whole tubers, unpeeled or peeled, in cross-sections or crushed in the form of puree [6,25]. However, these researchers have shown the potential of the NIR technology for the application in the potato industry. With the appearance of miniaturized or portable spectrometers, NIR spectral analysis became feasible directly in the field or during food processing [26,27]. The advance in the technological improvement of portable systems of NIR spectrometers is displacing the benchtop instruments, due to the advantages in the food industry [28]. In addition, this technique is favored by the increased availability of low-cost portable devices, which can be more easily implemented into the processing line. The estimation of potato quality parameters has not been applied with modern and portable systems of this type.

Currently, the food industry faces the challenge of the demand for high quality products with the possibility of monitoring much of the product in real time, but meeting with the requirements of food safety and traceability [29]. Spectroscopic sensors are optimal instruments for real time analysis of analytical techniques [27], with direct measurements in situ, which are very flexible and rugged, without the use of chemical reagents and waste [16,17,20,26,29]. The objective of this paper was to investigate the feasibility for measuring the main quality parameters of intact potatoes by means of a portable near-infrared (MicroNIR) spectroscopy device. The estimation of dry matter and reducing sugars content in potatoes was validated with NIR-spectra data and chemometrics.

## 2. Materials and Methods

### 2.1. Potato Samples

NIR recordings were performed directly on the tubers before chemical analysis in the laboratory. A superficial cleaning of the tubers was carried out to eliminate possible particles that could interfere with the spectral acquisition and obtain a representative sample of the whole tubers. The spectral and chemical measurements were carried out on six replicates (N = 534), resulting in an average value (N = 89) that was used for the subsequent chemometric treatments.

The sampled potatoes were grown during the crop seasons of 2019 and 2020 in A Limia region (Northwest of Spain). Two types of potato varieties were analyzed: Kennebec (N = 48) and Agria (N = 41). The choice of these types of potatoes is due to the different commercial destinations in this geographical region. Kennebec is intended for fresh consumption, and Agria for the potato processing industry. These potato cultivars are the ones with the highest production in the geographical area and are covered under the designation “PGI Patata de Galicia” recognized by the European Union.

### 2.2. Destructive Measurements of the Reference Quality Parameters

For reference analytical procedures, the tubers were gently washed to remove traces of soil adhering to the skin. Once the potatoes were dried, they were cut into four pieces for chemical analysis. Two alternate parts of the tuber were taken for the dry matter analysis by thermogravimetry, and the other two parts were used for the quantification of the reducing sugars content by a colorimetry method.

#### 2.2.1. Dry Matter Content

A piece of 5 g of each fresh potato was weighed to obtain the fresh weight (FW). Then, the sample cubes were placed in a dryer at 60 °C for 24 h. After this time, the samples were weighed to obtain the dry weight (DW). The dry matter content expressed in percentage was calculated according to Equation (1), based on the weight before and after drying.

Dry matter (%) = [(FW − DW)/FW] × 100(1)

#### 2.2.2. Reducing Sugars Content

The potato pieces selected for the determination of reducing sugars were crushed to form a puree. The potato solutions were prepared with 50 g of each potato puree dissolved in 200 g of distilled water. Then, 5 mL of the potassium oxalate solution (5%), 5 mL of the zinc acetate solution (0.1 M), and 5 mL of the potassium ferrocyanide solution (10.6%) were added to each mashed potato mixture (potato solution) to remove the reducing materials that were not sugars. At the same time, a blank solution was prepared. Thereafter, the potato and blank solutions were filtered. The oxidation of the reduced sugars was carried out with the ferricyanide solution. For this, 500 µL of the filtrated sample was deposited in a test tube with 10 mL of ferricyanide for 15 min in a boiling bath. The more intense the yellow color of the oxidation-reduction reaction, the greater the amount of ferricyanide remained unreacted, and the sample contained less reducing sugars. Finally, the intensity of the oxidation-reduction reaction of the solutions was measured by spectrometry at 422 nm at room temperature. Glucose solutions (0.2–0.8 g/L) as a reference standard were used for the calibration curve (R^2^ = 0.99). The reducing sugars content was expressed in g/100 g.

### 2.3. Near-Infrared Spectroscopy: Instrumentation and Spectral Data Acquisition

The NIR measurements of tuber samples were obtained using the portable MicroNIR Pro v2.5 equipment (MicroNIR 1700 ES, VIAVI, Santa Rosa, CA, USA) coupled to an instrument that is designed to measure the diffuse reflectance in the NIR region of the electromagnetic spectrum [30]. The portable MicroNIR system is easily handled and sized (45 mm diameter × 42 mm height; 60 g of weight), and it is equipped with a 128-pixel detector array [30]. The MicroNIR system employs a linear variable filter (LVF) as the dispersing element. The LVF is connected to a linear indium gallium arsenide (InGaAs array detector) into the equipment, which results in an extremely compact and rugged spectral engine with no moving parts [28]. Uncooled detectors of this type are often used since they offer good performance and cover the major part of the NIR spectral region [27,28]. The ultra-compact spectroscopic engine is coupled with a tungsten lamps diffuse illumination system.

**Figure 1:**
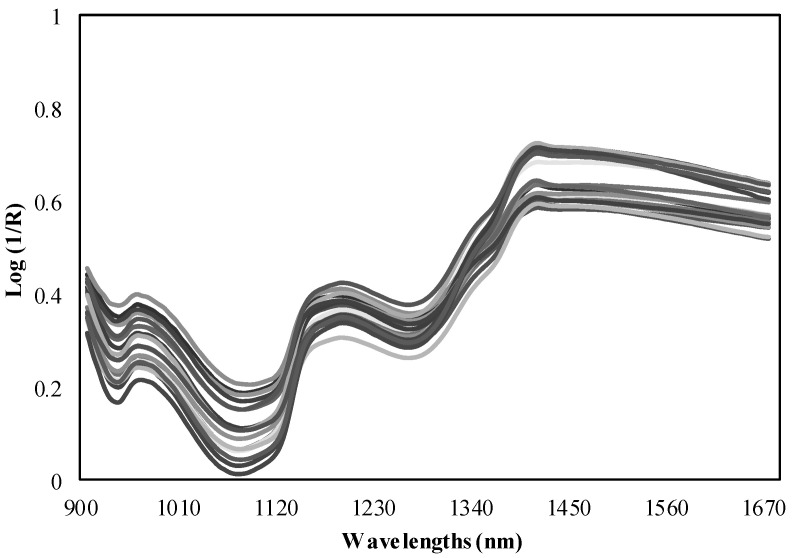
NIR spectra measured by the MicroNIR spectrometer.

NIR measurements were taken by the direct application with the MicroNIR spectrometer on tubers. Six replicate spectra were recorded for each sample and the average of the spectra was calculated (Figure 1). Spectra were recorded using the instrument acquisition software MicroNIR™ Pro v.2.2 (VIAVI, Santa Rosa, CA, USA) at intervals of 6 nm in the spectra in a range between 900–1700 nm. Spectral data were downloaded directly from the NIR equipment to a laptop connected through a USB port. However, this miniaturized spectrometer has the advantage of operating while it is connected by an USB interface to a tablet or wirelessly connected to a smartphone [28]. MicroNIR used a Spectralon^®^ ceramic tile as a white reference (100% reflectance) of polytetrafluoroethylene (~99%). The obtained spectra were combined into the spectral matrix, where the diffuse reflectance signal of the NIR spectrum is expressed as reflectance (R), using the values of log (1/R) for the chemometric analyses.

### 2.4. Chemometric Analysis

First, the data spectral matrix was subjected to principal component analysis (PCA) to perform the spectral selection of samples, maintaining the spectral variability of the original matrix. The methods of spectra pre-processing included the mathematical procedures for correction and improvement of spectra, which were applied before the qualitative and/or quantitative interpretation of spectral data [31]. The fluctuations or drift of the spectral baseline were reduced through normalization procedures as well as spectra derivation [27]. The applied pre-treatments to eliminate spectral dispersion effects were multiplicative dispersion correction (MSC), standard normal variant (SNV), DeTrend (DT) or SNV-DT [32]. The calibration for the quality parameters was obtained after removing the samples for spectral reasons, according to the Mahalanobis distance (H criterion = 3) and chemical reasons (T criterion ≥ 2.5) [20]. The mathematical treatments were also used to develop NIRS calibrations considering a code of four digits (for example, 1,4,4,1). This encoding explains the first digit as the number of the derivative, the second digit as the interval over which the derivative was calculated, the third as the number of data points in an average or smoothing, and the fourth as the second smoothing. Then, the samples were selected by this procedure to establish the calibration set, and the best treatment was chosen later to calibrate each quality parameter independently.

NIR models were developed using 89 potato samples: 70 samples for the calibration group and 19 samples for the external validation group. Partial least squares (PLS) regression was used to obtain the models with the best prediction performance, taking into account the different spectral pre-treatments. Calibration equations were performed by modified partial least squares regression (MPLS) using the raw spectral data and testing the different spectral treatments, as well as allocating the corresponding reference values to each sample. During the processing of this method, the cross-validation is recommended in order to select the optimum number of factors and to avoid overfitting [33]. The group of calibration samples is divided into a series of subsets in order to perform cross-validation. Then, each subset is validated with calibration, which is developed on the other samples [33]. Finally, several statistics were considered to evaluate the predictive capacity of the equations obtained. The standard error of cross-validation (SECV) is considered a good estimate for the prediction capability of the equation [33]. The ratio performance deviation (RPD) is a non-dimensional statistic for the evaluation of a NIR spectroscopy calibration model [34,35], which is the relation between the standard deviation of the reference chemical values (SD) and the root mean square error of prediction (SEP) in the NIR model and the standard error of cross-validation (SECV). The statistics used to select the best calibration equations were multiple correlation coefficients (RSQ) and the standard error of cross-validation (SECV). The software WinISI II v.1.50 (Infrasoft International, LLC, Silver Spring, MD, USA) was used for chemometric processing.

## 3. Results

### 3.1. Quantified Reference Data on Tubers: Dry Matter and Reducing Sugars

The descriptive analyses (mean, minimum, maximum, and relative standard deviation) of the dry matter and reducing sugars, which are quantified in the tubers are summarized in Table 1, according to the potato cultivar. The data were presented according to the two groups established for the NIR treatment: 70 samples constituted the denominated calibration group, and 19 samples were used for the validation group.

**Figure 2:**
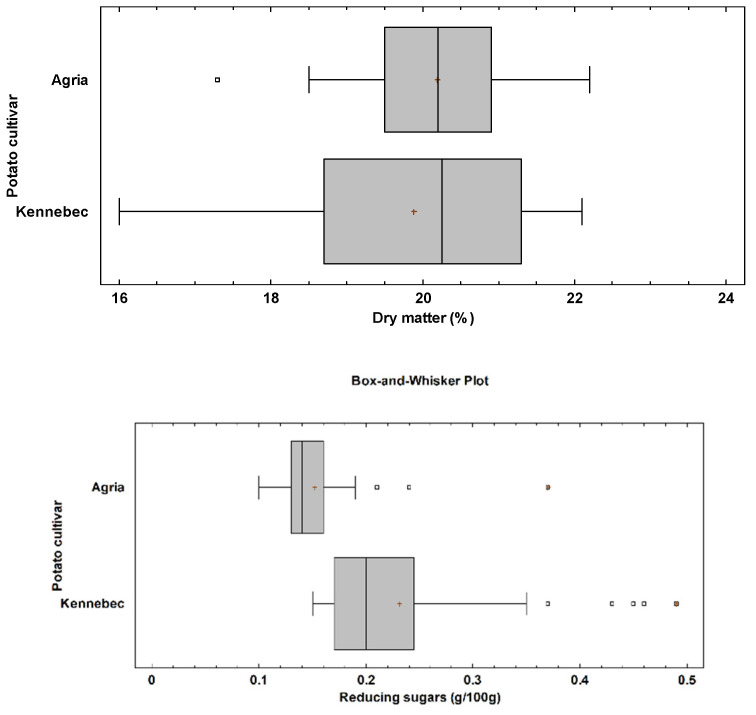
Box and whisker plot for dry matter (*p*-value = 0.68) and reducing sugars (*p*-value < 0.0001) by potato cultivar. The *p*-value according to the Kruskal-Wallis test by potato cultivar.

The mean dry matter content was similar between the tubers of Agria and Kennebec (*p* = 0.68) (Figure 2), with a mean value of 20.19% and 19.88%, respectively (Table 1). The box and whisker plot showed a higher range in the dry matter content of Kennebec tubers, with values between 16.0% and 22.1% (Figure 2), and a relative standard deviation of 1.63%. Regarding the reducing sugars, Kennebec cultivar had a significantly higher content than Agria (*p* < 0.0001), with a mean value of 0.23 g/100 g, and maximum value of 0.49 g/100 g (Figure 2). Agria tubers had a mean value and maximum value of 0.15 g/100 g and 0.37 g/100 g, respectively (Table 1). Therefore, the greater relative standard deviation in reducing sugars content in Kennebec tubers was found (0.09 g/100 g).

### 3.2. Spectral Information and NIR Calibration Equation

The PCA with the samples randomly selected in the calibration set was carried out (Table 1). The explained spectral variability was higher than 99.6% and between 4 and 10 principal components were required. In MPLS processing, the NIR residuals obtained after each factor and at each wavelength were calculated and standardized (dividing them by the standard deviations of the residuals at each wavelength) and then the next factor was calculated. The standardized method was conducted by dividing the NIR residuals with the standard deviations at each wavelength. Therefore, the obtained data of dry matter and reducing sugars, and the absorbance of the samples from 900 to 1700 nm were used to develop the calibration equations by this method.

The statistical parameters of calibration were obtained for each quality constituent after eliminating the samples using the spectral and chemical reasons. Between four and seven samples were eliminated to calibrate the dry matter, and seven and 10 samples for reducing sugars (Table 2). The optimal calibration equations for the determination of dry matter and reducing sugars were calculated based on the lowest SECV and the highest RSQ. The best NIR calibration models were shown in Table 2, indicating the best mathematical treatments, the range of applicability, the value of RSQ, and standard errors of calibration and cross-validation. For dry matter, the best equation showed a RSQ coefficient of 0.72 and a wide range of applicability (of the same order as the reference chemical method). Reducing sugars had a lower value of RSQ (0.55), and the marge of minimum and maximum values was acceptable. On the other hand, SEC and SECV were acceptable for both parameters. The RPD value was also taken into account to assess the predictive capacity of the models, with values of 1.90 and 1.48 for dry matter and reducing sugars, respectively (Table 2).

**Figure 3:**
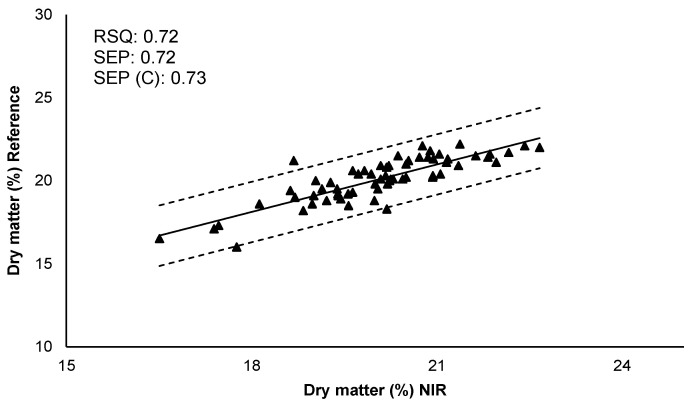

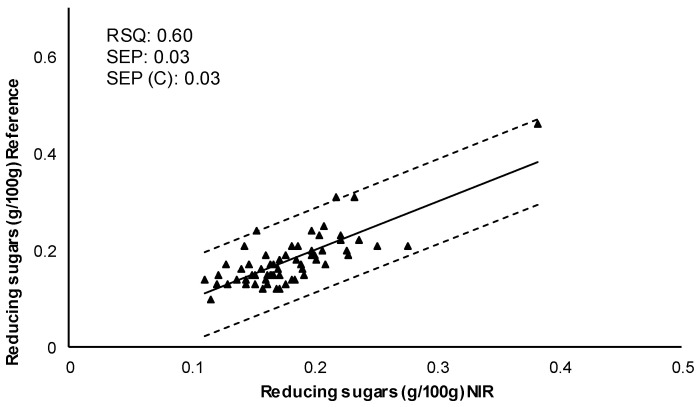
Internal validation. Comparison of reference values with predicted values by the NIRS model for each quality parameter. RSQ: Multiple correlation coefficient; SEP: Standard error of prediction; SEP (C): Standard error of prediction corrected by BIAS.

Cross-validation was carried out to evaluate the robustness of the obtained models. The set of calibration samples was divided into six subsets, of which five subsets were used for calibration and the other subset for the prediction set. This procedure was carried out several times, with the objective that all of the subsets were subjected to the calibration and prediction process. Subsequently, the resulting models for each parameter were validated and its predictive capacity was determined. The correlation between the values of reference (obtained by the reference method in laboratory) and the values predicted by NIR are represented in Figure 3. Internal validation showed better results for dry matter (higher RSQ and good errors of prediction) than reducing sugars. SEP, SEP (C), and bias indicated that the calibration models for the two quality parameters allows their determination. Therefore, the results reflected the prediction capacity and validity of the models. Although a larger number of samples including more types of potato cultivars, and more growing seasons could improve the predictions of these quality parameters.

### 3.3. External Validation and Prediction Capacity of the Models

The obtained calibration equations were validated with 19 new potato samples (validation set, Table 1) and the generated values were compared with the reference, according to the residual mean and root mean square error (RMSE) (Table 3). The predictive ability of models resulted as satisfactory, with RMSE of 1.17 and 0.07, and the mean residual values of 1.01 and 0.05 for dry matter and reducing sugars, respectively. The predicted values by NIR equations were compared with the reference data of samples that did not belong to the calibration equation using the Student’s t-test for paired values. The null hypothesis is accepted and there is no difference between the reference values and the NIR method generated for each parameter (*p* > 0.05).

## 4. Discussion

The demand for nutritional information and quality aspects of food by consumers makes it necessary to discover fast and safe methods that guarantee the safety and particularities of food. The precise assessment of potato freshness degree is a complex task. Food producers need techniques to evaluate changes in quality parameters, and non-destructive techniques, such as NIR technology, provide these advantages. These analytical methods are potentially useful tools to control the stability of the quality requirements in postharvest technology [3]. In this sense, the fresh and processed potato sector joins the challenge of offering quality products. Recent advances have shown good potentials of NIRS in real-time monitoring and modeling for different food processes. However, most of the studies have been carried out at a lab scale, while applications at industrial levels are still few [29], due to the difficulty of integrating scientific and industrial knowledge.

The sugars in potato tubers are very critical compounds for estimating the viability of processing, such as chipping and French frying [6,36]. In particular, glucose is responsible for the undesirable browning color of the frying process and it negatively affects the marketability of chips and other fried potato products [14]. PGI “Patata de Galicia” established values of reducing sugars content below 0.4% and dry matter above 18% for its industrial processing. Monitoring sugars in potato tubers before and during the storage has become a basic quality practice in the frying industry [6]. Therefore, it is of great importance for the processing industry that the quality of potato tubers meets their standards after storage [8].

In recent years, there have been investigations of the efficacy of NIR for determining dry matter and sugar contents in the potato processing industry. Benchtop NIR devices are generally built for experts in the laboratory, since they generate spectra that require interpretation and further data processing to generate a result. In response to an industrial demand, portable instruments can be designed for non-scientific personnel [28]. Portable NIR spectroscopic instrumentation and methods for spectral data analysis and interpretation are undergoing notable advancements. This has allowed for better optimization of the analytical procedures and the use of this technique directly on site [27].

NIR technology has been extensively studied for homogenized samples of potatoes, such as potato pulp, sliced potatoes, freeze-dried potato, and cooked potato mash [14,17,37,38], but to a lesser extent in intact potato [14,25,39,40]. Often, several pre-treatment methods are applied independently and the performance of the subsequent chemometric analysis was compared, in order to establish the best selection of the pre-processing set [27]. Regression analysis groups the methods used for the quantitative prediction of a physicochemical property for a large set of unknown samples. Then, the properly calibrated and validated regression model can be used significantly quicker and more efficiently compared with the conventional methods [27]. Therefore, chemometric methods are used to reduce the complexity of NIR spectral datasets and to build prediction models [17,20,37,41]. MPLS regression was used to estimate some chemical and quality constituents of potato tubers [14,17,25,40]. Specifically, models for glucose, sucrose, and soluble solids were built, with R^2^ in sliced potato samples (around 0.96, 0.83, and 0.50, respectively) higher than whole tubers (around 0.90, 0.80, and 0.30, respectively) [14]. The reducing sugars and dry matter content of potato varieties for frying (Innovator, Lady Claire, and Markies) according to three types of preparations (unpeeled, peeled, and transversally cut tubers) were compared, resulting in whole peeled potato tubers as the obtained maximum accuracy of the models to predict the dry matter (around R^2^ = 0.84) and reducing sugars (around R^2^ = 0.77) [25]. The best results in the estimation of dry matter concentration in sliced potato samples (R^2^ = 0.95) than the whole tuber (R^2^ = 0.85) were also found [39]. R^2^ around 0.98 was obtained for the prediction of dry matter content of the reported potato pulp [23]. In addition, lower values of R^2^ for glucose and fructose content on intact potato were obtained (with values of 0.65 and 0.71, respectively), as well as an acceptable standard error of prediction [40]. However, potato processors are more interested in determining the quality on whole tuber, but the application on intact unpeeled tubers is less frequent.

The accuracy and goodness of the models were evaluated according to the statistic RPD. Some researchers considered that a RPD ratio of less than 1.5 indicates poor predictions and a value higher than 2 indicates a good calibration [20]. Although the results in the present study using the portable equipment were not excellent, in the case of dry matter they were acceptable (RDP = 1.98). The calibration was performed on unpeeled potato tubers, which complicates the model robustness of these quality parameters. In addition, it must be taken into account that products with a low moisture content, such as ground or dehydrated samples, usually presented high values of RPD. This is the case of the estimation of dry matter and total soluble solid content on peeled and freeze-dried potato by the NIR system, with a RPD greater than 2.8 [17]. For the prediction of glucose content on whole tubers, a RDP of around 2 was reported [14], and for reducing sugars a RPD lower than 2 was found [40]. Sugars are generally reported to yield a lower prediction performance by NIR technology [6,37,38]. Some researchers mentioned the effect of the skin on the lower performance of the models that varies depending on the cultivar [6]. Consequently, sorting potato tubers based on sugar content is a more challenging task than assessing sugars in ground, homogenized or even sliced samples. In particular, the peeled potato was determined as the most interesting in order to obtain precise models for sugar and dry matter contents, which improves the RPD values from 15% to 38% for reducing sugars and 35% for dry matter [25]. However, potato processors are interested in obtaining the prediction models for whole tubers, in order for the technology to be developed for application in potato processing lines.

The calibration models should be based on large datasets, which are obtained from different destinations, growing conditions, and operational conditions [18]. This is the first study to apply the MicroNIR directly to whole tubers in an area closely linked to the potato crop and with a notable economic impact on the agricultural sector of the Galician community. Rapid measurement devices, such as the MicroNIR, calibrated for working with multiple cultivars and different shapes make its application attractive for the potato industries. The portable equipment incorporated the analytical precision required for chemical identification and quantification with a spectral resolution, which is equivalent to the benchtop instruments [26,27]. These portable devices have the advantages of ease of transportation and the necessary flexibility for an analysis in an industrial environment. Undoubtedly, technological innovations in portable instruments have been increased by developing interesting advantages in their application in-line with respect to laboratory equipment.

## 5. Conclusions

Taking into account the industrial range defined for reducing sugars and dry matter parameters according to the PGI Patata de Galicia standard, we consider the MicroNIR as a useful portable device and with a promising performance for the sector. The efficacy of NIR spectroscopy was demonstrated as a rapid and non-destructive method for the estimation of dry matter and reducing sugars in intact potato tuber. Although the model calibrations were not excellent, as a first approach for the use of this technology on-site in the potato warehouses, it is attractive for the sector. The results are interesting for the use of MicroNIR as a tool for fresh potato quality control during in-line processing. The efficiency of the automation techniques of this type optimizes the management of industrial processing, guaranteeing the quality of the potato tubers. However, a close collaboration between the potato processors and researchers is very important in order to achieve these goals and for future improvements.

## Figures and Tables

**Table 1:** An overview of the samples by potato cultivar and distribution by the calibration and validation set.

		Mean	SD	Min	Max
Samples set by potato cultivar					
Kennebec (N = 48)	Dry matter (%)	19.88	1.63	16.00	22.10
	Reducing sugar (g/100 g)	0.23	0.09	0.15	0.49
Agria (N = 41)	Dry matter (%)	20.19	1.04	17.30	22.20
	Reducing sugar (g/100 g)	0.15	0.04	0.10	0.37
Calibration set (N = 70)					
	Dry matter (%)	19.67	2.07	16.0	22.0
	Reducing sugar (g/100 g)	0.19	0.08	0.10	0.49
Validation set (N = 19)					
	Dry matter (%)	19.89	1.25	17.80	22.0
	Reducing sugar (g/100 g)	0.20	0.09	0.12	0.43
Total sample set (N = 89)					
	Dry matter (%)	20.03	1.39	16.00	22.20
	Reducing sugar (g/100 g)	0.19	0.08	0.10	0.49

SD: Relative standard deviation; Max: Maximum; Min: Minimum.

**Table 2:** Calibration descriptors of the best models obtained for each parameter by NIR.

Constituent	Math Treatment *	N	Mean	SD	Range of Applicability		SEC	RSQ	SECV	RPD
					Min	Max				
Dry matter	Detrend only 0,0,1,1	65	20.09	1.36	16.00	24.18	0.72	0.72	0.93	1.90
	Standard MSC 2,10,10,1	65	20.04	1.42	15.77	24.31	0.75	0.72	10.21	1.89
	None 2,4,4,1	65	20.17	1.25	16.41	23.94	0.68	0.71	0.98	1.85
	Standard MSC 2,4,4,1	63	20.15	1.28	16.30	24.00	0.70	0.70	0.96	1.84
	SNV only 2,4,4,1	66	20.07	1.43	15.76	24.37	0.79	0.70	10.31	1.82
Reducing sugars	SNV only 0,0,1,1	62	0.18	0.06	0.01	0.35	0.04	0.55	0.05	1.48
	Detrend only 2,8,6,1	61	0.17	0.04	0.06	0.28	0.02	0.51	0.03	1.42
	Standard MSC 0,0,1,1	63	0.17	0.04	0.05	0.29	0.03	0.50	0.03	1.41
	Detrend only 0,0,1,1	61	0.17	0.04	0.06	0.28	0.03	0.48	0.03	1.39
	None 2,4,4,1	60	0.17	0.04	0.06	0.27	0.03	0.48	0.03	1.39

N: Number of samples after removing the outliers; MSC: Multiplicative dispersion correction; SNV: Standard normal variate; SD: Standard deviation; Min: Minimum; Max: Maximum; RSQ: Multiple correlation coefficients; SEC: Standard error of calibration; SECV: Standard error of cross-validation; RPD: Ratio performance deviation. * In the math treatment, the first digit is the number of the derivative, the second is the gap over which the derivative is calculated, the third is the number of data points in a running average or smoothing, and the fourth is the second smoothing.

**Table 3:** External validation (19 samples) of potato quality parameters with the results of the NIR calibration.

Constituent	Mean Residual	RMSE	*p*-Value
Dry matter	1.01	1.17	0.22
Reducing sugars	0.05	0.07	0.16

RMSE: Root mean standard error. *p*-value: Level of significance calculated according to the Student’s test.

## Data Availability

Data is contained within the article.
